# Physiological Basis of Heterosis for Nitrogen Use Efficiency of Maize

**DOI:** 10.1038/s41598-019-54864-x

**Published:** 2019-12-10

**Authors:** Zhigang Wang, Bao-Luo Ma, Xiaofang Yu, Julin Gao, Jiying Sun, Zhijun Su, Shaobo Yu

**Affiliations:** 1 College of Agronomy, Inner Mongolia Agricultural University, Hohhot, 010019 China; 2 Ottawa Research and Development Centre, Agriculture and Agri-Food Canada, Ottawa, ON K1A 0C6 Canada

**Keywords:** Plant breeding, Plant breeding, Plant physiology, Plant physiology

## Abstract

Efficient use of nitrogen inputs for concurrent improvements in grain yield and nitrogen use efficiency (NUE) has been recognized as a viable strategy for sustainable agriculture development. Yet, there is little research on the possible physiological basis of maize hybrid heterosis for NUE and measurable traits that are corresponding to the NUE heterosis. A field study was conducted for two years to evaluate the heterosis for NUE and determine the relationship between NUE and its physiological components. Two commercial hybrids, ‘Xianyu335’ and ‘Zhengdan958’, and their parental inbred lines, were grown at 0 (0 N) and 150 kg N ha^−1^ (150 N), in a randomized complete block design with four replications each year. Compared to their parental lines, both hybrids displayed a significant heterosis, up to 466%, for NUE. N internal efficiency (NIE) accounted for 52% of the variation in heterosis for NUE, while there was generally negligible heterosis for nitrogen recovery efficiency (NRE). Heterosis for NIE and thereby for NUE in maize was ascribed to (i) an earlier establishment of pre-anthesis source for N accumulation, which phenotypically exhibited as a faster leaf appearance rate with higher maximum LAI and photosynthetic nitrogen use efficiency; (ii) a larger amount of N being remobilized from the vegetative tissues, especially from leaves, during the grain filling. Phenotypically, there was notably a rapid reduction in post-anthesis specific weights of leaf and stalk, but with maintained functionally stay-green ear leaves; and (iii) a higher productive efficiency per unit grain N, which was characterized by a reduced grain N concentration and enhanced sink strength.

## Introduction

The global challenge of meeting increased food demand and protecting environmental quality calls for improving nitrogen use efficiency in maize production systems. Exploring maize grain yields with minimum nitrogen inputs is an inevitable choice in the development of sustainable agriculture^1^. Exploring maize biological potential for nitrogen use efficiency (NUE) has been recognized as an important approach to achieve simultaneously improvements in maize yield, and environmental quality^2^.

Nitrogen use efficiency in system level of crops is usually expressed by agronomic efficiency (AE) of the applied N, i.e. yield increment per unit N applied^2^. From an agronomic point of view, NUE in maize can be partitioned into nitrogen recovery efficiency (NRE) and nitrogen internal efficiency (NIE) (NUE = NRE × NIE)^3^. NRE reflects the ability of aboveground plant parts to recover N from the applied N fertilizer, and NIE reflects the capability of plants to transform the N taken up by the crop into grain^4^. Crop NUE is closely related to the accumulation and allocation of plant biomass^5^ and nitrogen^6–8^. Evaluation of the two components is useful to advance the understanding of physiological mechanisms of NUE. This is achieved through examining N uptake, assimilation, translocation and remobilization^3^. To achieve high NUE, some authors have observed that the NRE component was more important under high N supply environments; whereas the NIE component was more essential in low N availability environments^9,10^. Ciampitti and Vyn^11^ reviewed the physiological mechanisms of changes over time in maize NUE. They suggested that New Era genotypes, which exhibited a higher NUE, showed higher total biomass (BM) accumulation and N uptake during the post-silking period. Higher NUE of New Era genotypes were primarily associated with the reduced grain %N and NIE. Ma and Dwyer^12^ and Subedi & Ma^13,14^ also suggested that hybrids with greater yields or higher NUE were accompanied with greater BM production and more N uptake during the grain-filling period (i.e. high NRE), but their results showed no indication of greater allocation of N to the grain in hybrids with higher yields or NUE (i.e. low NIE)^13^. Thus, there appears a trade-off relationship between NRE and NIE. Wang, *et al*.^15^ suggested that NRE was positively associated with root mass at silking and with post-anthesis N accumulation, while NIE seemed to rely on the ratio of dry matter accumulation pre-anthesis to post-anthesis. Maize plant nitrogen uptake and NUE are closely related to mid-season field phenotypes. Ciampitti, *et al*.^16^ found that leaf chlorophyll (SPAD readings) and plant stem volume at silking stage were good indicators of final plant N uptake, grain yield and NUE of maize. At molecular level, location of QTLs for N remobilization and N uptake had been detected. Hirel *et al*.^17^ also observed coincidences between QTLs for leaf glutamine synthetase (GS) activities and NUE traits (nitrogen remobilization, and post-anthesis nitrogen uptake), they proposed that leaf nitrate accumulation and the reactions catalyzed by nitrate reductase (NR) and GS are coregulated and represent key elements controlling NUE for grain filling in maize. However, isolating gene(s) involved in the expression of NUE traits has not been achieved because of the lack of a reliable phenotypic screen system.

Heterosis of a maize hybrid is described as the mid-parent heterosis (MPH) (by measuring its superior dry matter, HI and grain yield performance) over the mean performance of its parental inbred lines. During the past decades, the physiological basis of heterosis for maize yield has been systematically illustrated and documented^18–21^. Heterosis for grain yield in maize was attributed to heterosis for leaf size, maximum LAI, leaf stay-green traits, sustaining rate of photosynthesis during the grain filling period and larger harvest index (HI)^18^. Increasing plant density did not affect heterosis for dry matter at maturity, but caused an increase in heterosis for grain yield and HI. The increased heterosis for HI under increasing plant density was associated with a greater plant-to-plant variability and a higher threshold plant dry matter for HI at maturity in the inbred lines as compared to the hybrid^20^. Shading stress during the presilking and silking periods also resulted in a greater heterosis of maize hybrids for grain yield, which was highly associated with heterosis for kernel number. Heterosis for kernel set was attributable to the heterosis for the relative growth rate (RGR) during the one-month period bracketing silking^19^.

In maize hybrid breeding programs, plant breeders usually focus on the utilization of heterosis for grain yield and its related phenotypes^22^. The utilization potential of heterosis for maize NUE has recently been reported^23^. However, previous studies focused on the outcome of heterosis for maize NUE without examining the processes of the possible physiological basis and the components leading to NUE heterosis. There was a lack of fundamental understanding of NUE heterosis, and there were no reliable and measurable traits that serve as indicators of NUE heterosis available for selection. It is of crucial importance to illustrate the physiological basis underlying the heterosis of maize NUE for simultaneously enhancing yield and NUE through cultivar improvement and/or management strategies. Therefore, a field experiment was conducted on two contrasting commercial maize hybrids and their parental inbred lines, to investigate yield, NUE, N uptake and remobilization, and the related traits. Our objective was to illuminate the main physiological basis of heterosis for maize NUE.

## Results

### Heterosis for nitrogen use efficiency and its component processes

Mean values of the hybrids and inbred lines differed significantly for most of the variables, except for nitrogen recovery efficiency, shoot N concentration, and NHI (Table 1). During the two study years, NUE of hybrids ranged from 21.1 to 30.7 kg grain kg^−1^ N applied, compared to 5.9 to 8.0 kg grain kg^−1^ N applied for their inbred parents, indicating a significant heterosis for NUE. On average, there exhibited a mid-parental heterotic NUE response of 422% in 2014, and 174% in 2015, with ranges from 141% to 783%. This difference indicated a large impact of environments on maize heterosis for NUE (Table 2). There were also large variations among the physiological parameters related to NUE. Specifically, NIE exhibited a mid-parental heterotic response of 218% in 2014 and 132% in 2015, while there was a minimal heterosis for NRE, compared to the inbred lines.

**Table 1:** Nitrogen use efficiency, grain yield and the related components of maize hybrids and their parental inbred lines at different nitrogen rates in 2014 and 2015.

Variable^†^	Unit	2014				2015			
		Hybrid		Lines		Hybrid		Lines	
		0 N	150 N	0 N	150 N	0 N	150 N	0 N	150 N
NUE	kg grain kg^−1^ N applied	—	30.7a^‡^	—	5.9b	—	21.1a	—	8.0b
NIE	kg grain kg^−1^ N uptake	—	78.5a	—	24.7b	—	56.9a	—	25.0b
NRE	kg N kg^−1^ N applied	—	0.4a	—	0.4a	—	0.4a	—	0.4a
Plant N	kg N ha^−1^	221.5b	284.4a	132.4c	194.6b	187.1b	244.0a	126.5c	183.4b
Grain N	kg N ha^−1^	151.1 b	208.9a	93.7d	142.4c	122.5b	172.4a	83.1d	113.1c
Remobilized N	kg N ha^−1^	67.9c	140.5a	66.6c	80.5b	51.0b	85.5a	47.4b	33.5c
RLN	kg N ha^−1^	35.8b	77.7a	27.1b	42.7b	44.6b	52.4a	30.8c	24.6d
RSN	kg N ha^−1^	32.1b	62.8a	39.5b	37.8b	24.0b	28.2a	16.6c	13.2c
Vegetative N	kg N ha^−1^	138.3b	216.0a	105.4c	132.7b	115.6b	157.2a	90.8c	103.8b
Reproductive N	kg N ha^−1^	72.2a	85.5a	27.0b	76.9a	71.6a	86.8a	46.0b	75.9a
NRR	%	50.8a	59.8 a	63.4a	61.4a	44.1b	53.2a	49.5b	31.5c
NCR	%	45.1c	66.9a	70.5a	59.1b	46.7b	48.5b	55.8a	29.4c
Grain %N	%	1.3b	1.4a	1.2b	1.6a	1.3b	1.5b	1.7a	1.9a
Shoot %N	%	0.8a	0.8a	0.7a	0.7a	0.7a	0.8a	0.8a	1.0a
NHI	kg kg^−1^	0.7a	0.7a	0.7a	0.7a	0.6a	0.7a	0.7a	0.6a
PNUE	μmol mol^−1^ s^−1^	270.3b	415.1a	157.3d	188.1c	222.0b	277.5a	154.6d	202.4c
GY	Mg ha^−1^	9.0c	13.6a	7.0b	7.9bc	10.1b	13.3a	5.9d	7.1c

^†^NUE, nitrogen use efficiency; NIE, nitrogen internal efficiency; NRE, nitrogen recovery efficiency; Plant N, plant N uptake at physiological maturity per-unit-area; Grain N, grain N uptake at physiological maturity per-unit-area; Remobilized N, reproductive-stage shoot N remobilization; RLN, reproductive-stage leaf N remobilization; RSN, reproductive-stage stalk N remobilization; Vegetative N, vegetative-stage whole-plant N uptake; Reproductive N, reproductive-stage whole-plant N uptake; NRR, shoot N remobilization ratio; NCR, contribution ratio of shoot remobilization N to grain N; Grain %N, grain N concentration at physiological maturity; Shoot %N, shoot N concentration at physiological maturity; NHI, nitrogen harvest index; PNUE, photosynthetic nitrogen use efficiency; GY, grain yield per-unit-area. The same below.

^‡^Values of the same variable within the same year followed by different letters are significantly different according to an ANOVA-protected LSD 0.05 test.

**Table 2:** Absolute heterosis (AH) value, mid-parental heterosis (MPH) value and range of mid-parental heterosis for nitrogen use efficiency and the related parameters of maize hybrids.

Variable	2014			2015		
	AH	MPH	Range of MPH	AH	MPH	Range of MPH
		%			%	
NUE	24.8**	421.7**	230.6–782.9	13.1**	173.5**	140.9–366.4
NIE	53.8**	218.3**	93.5–373.5	31.9**	132.1**	45.1–250.8
NRE	0.01 ns^†^	2.5 ns	(−43.6)–64.5	0.01 ns	0.55 ns	(−31.9)–54.7
Plant N	89.8**	46.1**	25.2–99.6	60.5**	33.3**	6.1–83.4
Grain N	66.5**	46.7**	24.8–77.9	59.3**	52.7**	24.5–66.9
Remobilized N	59.9*	74.4*	(−38.7)–86.3	52.0*	168.9*	(−10.9)–198.1
RLN	35.0*	81.8*	(−13.1)–1138.1	27.8*	127.0*	68.7–180.4
RSN	25.0*	66.0*	(−83.1)–151.1	15.0*	127.0*	(−77.3)–339.5
Vegetative N	83.3**	62.7**	24.2–69.6	53.3**	52.0**	(−1.7)–96.4
Reproductive N	8.6 ns	11.2 ns	10.2–12.0	10.9 ns	14.4 ns	6.0–29.8
NRR	1.6 ns	2.7 ns	(−50.1)–21	22.0*	74.4*	(−39.1)–82.1
NCR	7.8*	13.1*	(−62.1)–50.4	19.1*	68.0*	(−40.1)–126.0
Grain %N	0.1 ns	−5.2 ns	(−13.1)–18.1	−0.4**	−19.1**	(−46.1)–(−12.9)
Shoot %N	0.1 ns	18.8 ns	(−33.4)–219.0	−0.2 ns	−19.4 ns	(−60.4)–144.8
NHI	0.0 ns	0.1 ns	(−21.3)–26.6	0.1 ns	15.1 ns	(−13.4)–18.9
PNUE	227.0**	120.7**	71–127	75.1*	39.0*	18–181
GY	5.7**	73.1**	9.5–95.8	6.2**	86.7**	50.4–106.9

^†^Ns, not significant; *, **, significant at the 0.05 or 0.01 level of probabilities.

**Figure 1:**
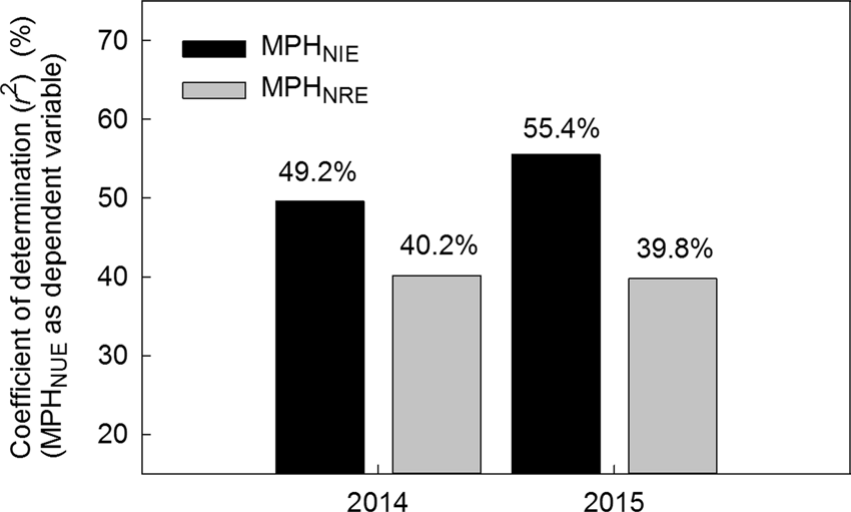
Coefficients of determination (r^2^) of mid-parental heterosis for NUE (MPH_NUE_) (dependent variable) versus mid-parental heterosis for NRE (MPH_NRE_) and mid-parental heterosis for NIE (MPH_NIE_) in 2014 and 2015.

To examine the respective contribution of NRE and NIE to the MPH for NUE, a linear regression was conducted between the MPH values of NUE and its two components. The coefficients of determination (r^2^) between MPH_NUE_ and MPH_NIE_ were 49.2% in 2014 and 55.4% in 2015, compared to 40.2% and 39.8% for MPH_NRE_ (Fig. 1). The closer association of MPH_NUE_ with MPH_NIE_ (r = 0.95, p < 0.01) than with MPH_NRE_ (r = 0.35, p > 0.05) was a clear indication of the primary role of NIE in heterosis for NUE.

N uptake, translocation and remobilization related variables such as plant N, grain N, remobilized N (including remobilized leaf N and remobilized stalk N), vegetative N, N remobilization ratio (NRR), N contribution ratio (NCR) and photosynthetic nitrogen use efficiency (PNUE), all showed significant positive heterotic effects, and were positively correlated with MPH_NIE_. Reproductive N, grain N concentration, shoot N concentration and NHI did not exhibit obvious heterosis (Table 2). It is noticeable that grain N concentration exhibited a negative heterotic effect in both years, meaning lower grain N concentrations of the hybrids than of their parental inbred lines. As expected, grain yield and its related components all exhibited significant heterosis. The magnitude of the heterotic effect varied greatly across the physiological parameters as presented above, with the heterotic responses ranging from −83.1% to 1138.1% (Table 2). This inconsistency in heterotic effects indicates differential responses of these physiological traits and processes in determining NUE and/or NIE between inbred lines and the hybrids.

### Physiological basis underlying the heterosis for nitrogen use efficiency and its component processes

**Figure 2:**
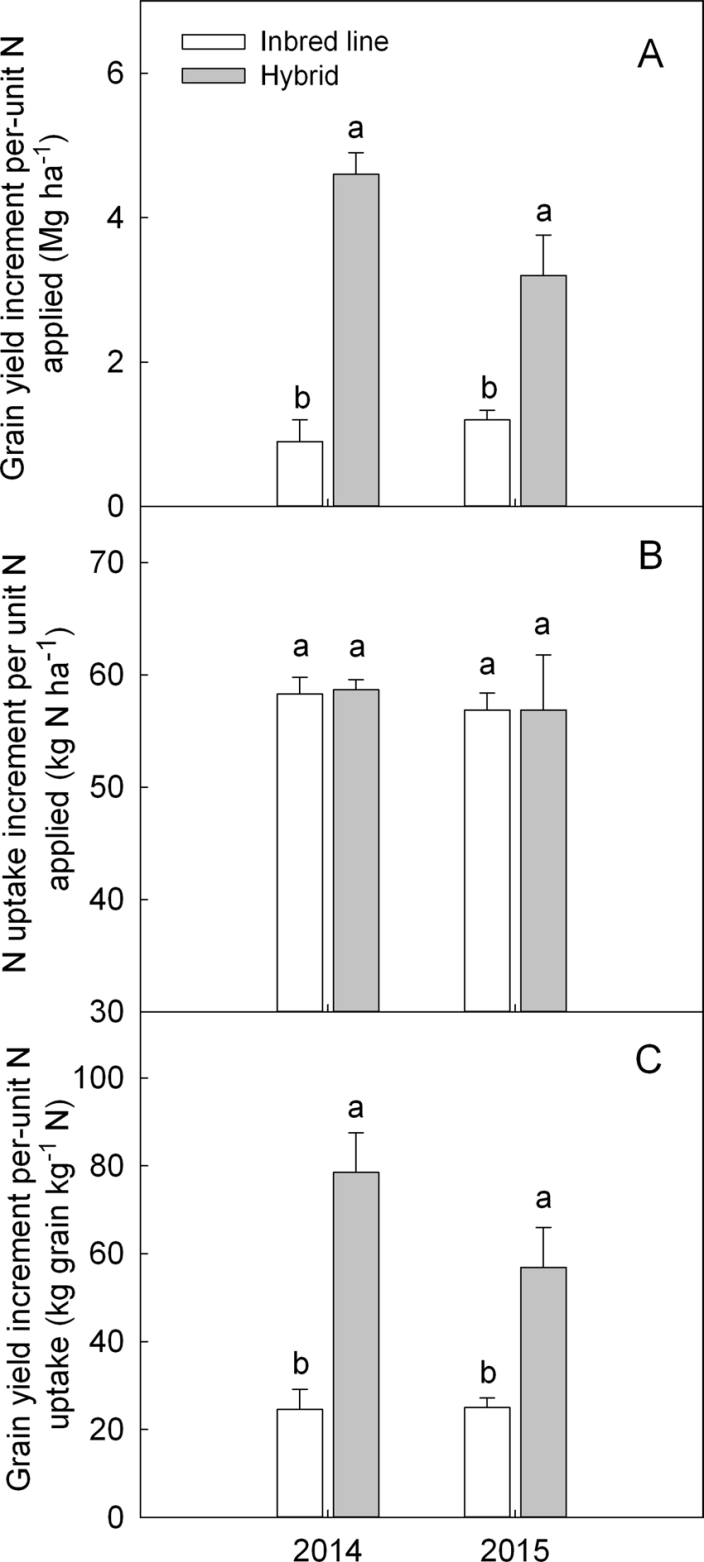
Comparison of grain yield increment per-unit N applied, N uptake increment per unit N applied and grain yield increment per-unit N uptake of hybrids and parental inbred lines of maize in 2014 and 2015. Means followed by different letters indicate significant differences according to an ANOVA-protected LSD_0.05_ test.

Grain yield increment per-unit N applied was 411% in 2014 and 167% in 2015, respectively greater for hybrids than for the inbred lines (Fig. 2A). However, the N uptake increment per-unit N applied was similar between the hybrids and inbred lines, even though the absolute N uptake was obviously greater for the hybrids than for inbred lines under different N rates (Fig. 2B). In this case, hybrids showed greater grain yield increment per-unit N uptake by 219% in 2014 and 128% in 2015, respectively than that of the inbred lines (Fig. 2C). Therefore, hybrids exhibited significant heterosis for NUE and NIE, but not for NRE.

**Figure 3:**
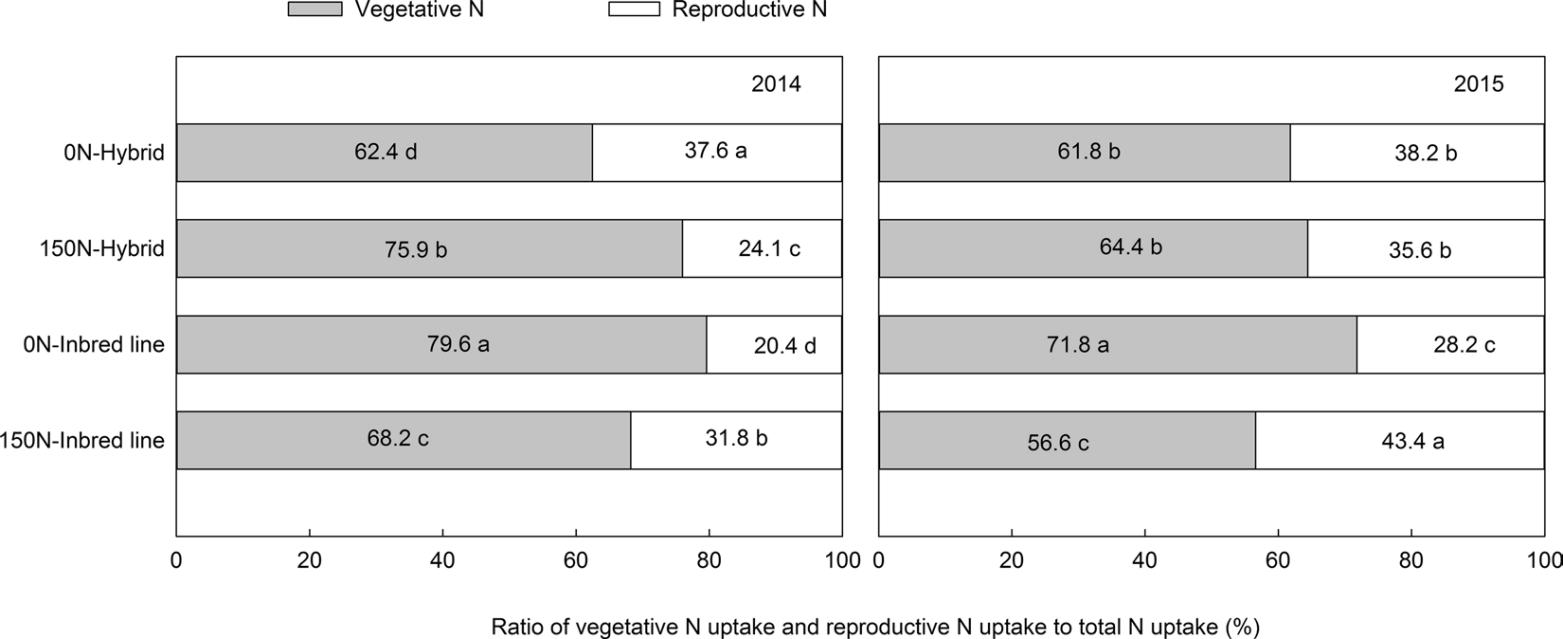
Ratio of vegetative N uptake and reproductive N uptake to total N uptake of maize hybrids and parental inbred lines under different nitrogen rates. Means in the same growth stage within a year followed by different letters indicate significant differences according to an ANOVA-protected LSD_0.05_ test.

**Figure 4:**
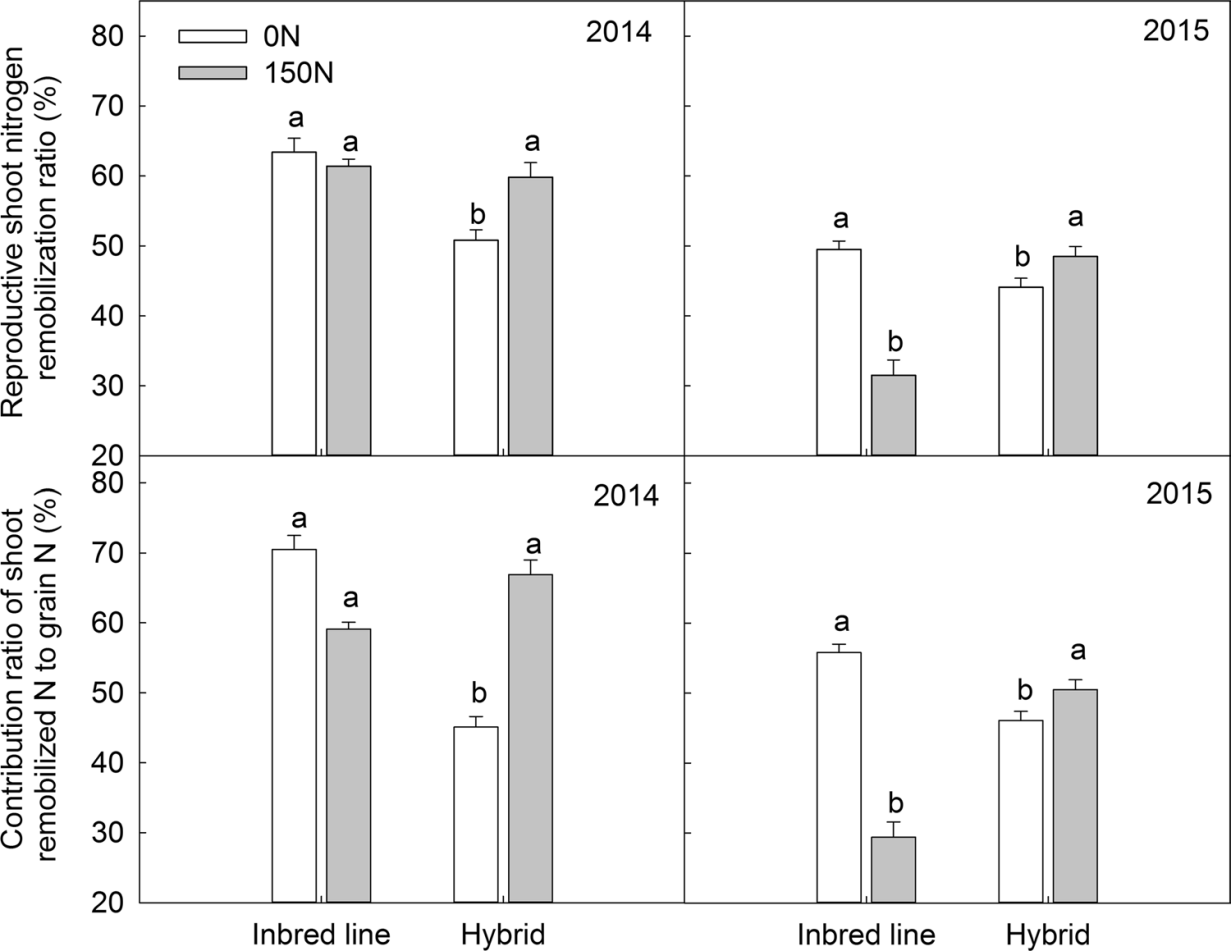
Nitrogen remobilization ratio (NRR) of maize hybrids and their parental inbred lines under different nitrogen rates in 2014 (left) and 2015. Means in the same year followed by different letters indicate significant differences according to an ANOVA-protected LSD_0.05_ test.

**Figure 5:**
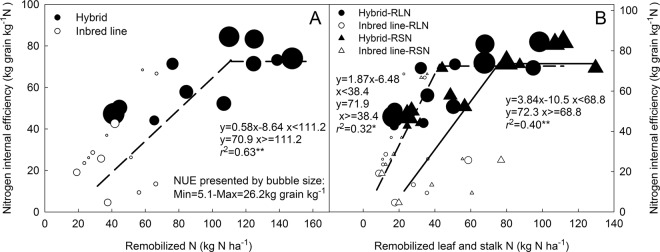
Relationship between nitrogen internal efficiency and shoot remobilized nitrogen (**A**) and remobilized leaf and stalk nitrogen (**B**) of hybrids and parental inbred lines.

The applied N treatment significantly increased the ratio of vegetative N uptake to total N uptake in the hybrids, but this ratio was decreased by 11.4% and 15.2% respectively in their parental inbreds (Fig. 3). This indicates that hybrids exhibited great heterotic effect on vegetative N uptake, an important base for N remobilization during the reproductive stage. Shoot N remobilization of the hybrids was improved through an increased N remobilization ratio (NRR) from 50.8% (0 N) to 59.8% (150 N) in 2014 and from 44.1% to 53.2% in 2015. In comparison, NRR of the inbreds decreased from 63.4% to 61.4% in 2014 and from 49.5% to 31.5% in 2015 (Fig. 4). This directly influenced the contribution ratio of shoot remobilized N to grain N. Similarly, the applied N treatment increased NCR in hybrids by up to 67% in 2014 and by approximately 47% in 2015. In contrast, NCR in the inbred parents was decreased by the N application. This explained the obvious heterosis for NRR and NCR of the hybrids. Consequently, an average of 113 kg N ha^−1^ was remobilized during the reproductive stage in the hybrids, which was twice as much of that (57 kg N ha^−1^ in average) in the inbred lines (Fig. 5).

**Figure 6:**
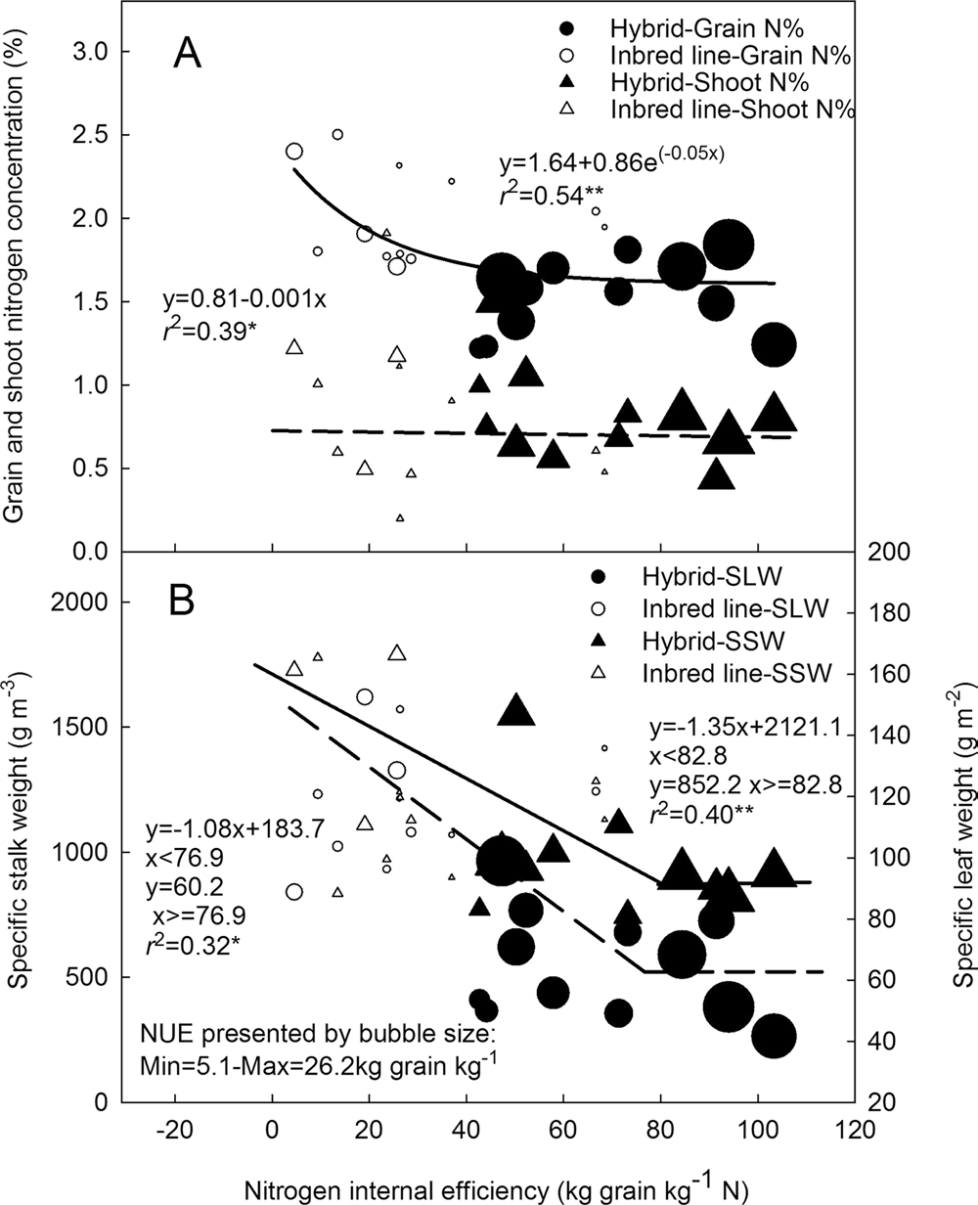
Relationship of nitrogen internal efficiency with shoot %N and grain %N (**A**) at physiological maturity, and with specific leaf weight and specific stalk weight (**B**) at physiological maturity of hybrids (black color) and parent inbred lines (white color).

The relationship between the reproductive shoot remobilized N and NIE was best represented by the linear-plateau model when the data of inbred lines and hybrids was pooled together. The NIE value increased initially with increasing remobilized N, and plateaued at approximately 70 kg grain kg^−1^ N. The remobilized N reached its peak value of 111.2 kg N ha^−1^, which represented the potential of NIE and the corresponding amount of remobilized N needed for achieving the NIE (Fig. 5A). Because the remobilized N was primarily derived from the leaf and stalk, a linear-plateau model also well fitted the relationship of NIE, respectively with the remobilized leaf N (RLN) and the remobilized stalk N (RSN) (Fig. 5B). RLN and RSN were significantly higher for hybrids than for the inbred lines. The greater RLN than RSN (32.1% on average) indicates a more important role of leaf N remobilization in NIE enhancement during the grain filling. NIE was negatively related with the grain %N (r = −0.58, p < 0.05), but unrelated with shoot %N (Fig. 6A). While with similar shoot %N, grain %N was 28% lower in hybrids than in their parent inbred lines, which was one of the main reasons for heterosis for NIE in maize hybrids.

### Physiological basis underlying the heterosis for nitrogen use efficiency related phenotyping

Plant phenotypic traits related to N uptake, N distribution and N remobilization were remarkably enhanced in hybrids than in inbred lines under the applied N versus 0 N application conditions (Table 3). The applied N treatment accelerated leaf appearance rate (RLA_V6-R1_) of the hybrids by 13% to 23%, compared to 10% to 15% improvement in the inbred lines. At silking, the increments of maximum LAI (LAI_R1_) and leaf chlorophyll content (SPAD_R1_) by N application were all greater for the hybrids than for the inbred lines. N application also enhanced photosynthetic nitrogen use efficiency (PNUE) by up to 54% in the hybrids, compared to, at the most, 31% in the inbred lines (Table 1). Clearly, a strong heterotic effect on leaf growth was the basis of the heterosis for vegetative N uptake in maze hybrids.

**Table 3:** Phenotypic characters for nitrogen uptake and/or nitrogen remobilization of hybrids and parental inbred lines under different nitrogen rates in 2014 and 2015.

Variable^†^	Unit	2014				2015			
		Hybrids		Inbred lines		Hybrids		Inbred lines	
		0 N	150 N	0 N	150 N	0 N	150 N	0 N	150 N
LAI_R1_	m^2^ m^−2^	3.4 b^‡^	3.7a	1.9c	1.9c	3.2b	3.8a	1.8d	1.9c
LAI_R6_	m^2^ m^−2^	0.8b	0.9a	1.0 b	0.9b	1.0a	0.9a	1.0 a	1.0a
SPAD_R1_		47.6b	55.9a	45.7 c	47.2b	46.7b	57.6a	44.6c	47.2bc
SPAD_R6_		21.4b	26.7a	16.1 c	14.6d	21.1b	25.9a	13.4 d	13.1c
Pn	μmol·m^−2^·s^−1^	16.3b	21.0a	10.5c	16.9b	20.9b	24.5a	14.5c	16.1c
RLA_V6-R1_	leaves d^−1^	0.31bc	0.35a	0.30c	0.33b	0.30b	0.37a	0.27c	0.31b
SSW	g m^−3^	904b	680d	865c	1207a	1020b	711d	893c	1217a
SLW	g m^−2^	97.2c	88.3b	121.2b	133.5a	58.0c	61.1d	85.3a	94.0a

^†^LAI_R1_, LAI at silking stage; LAI_R6_, LAI at physiological maturity; SPAD_R1_, SPAD value at silking stage; SPAD_R6_, SPAD value at physiological maturity; Pn, leaf apparent photosynthetic rate; RLA_V6-R1_, leaf appearance rate from 6 leaf collar stage to silking stage; SSW, specific stalk weight; SLW, specific leaf weight.

^‡^Values of the same variable within the same year followed by different letters are significantly different according to an ANOVA-protected LSD 0.05 test.

During the reproductive stage, there was a greater reduction in LAI for hybrids (75.7% to 76.3%) than for the inbred lines (47.4% to 52.6%). However, the hybrids maintained larger chlorophyll values of the ear-leaves than the inbreds. This indicates that efficient N remobilization induced a quicker leaf area recession at the bottom and the top of the plants. By keeping longer stay-green and higher chlorophyll concentration of ear leaves, hybrids had a higher photosynthetic nitrogen assimilation than the inbred lines. This supported the heterotic effect on PNUE.

Biomass and N remobilization induced a reduction in specific leaf weight (SLW) and specific stalk weight (SSW). We observed a negative correlation of NIE with SLW, and with SSW (Fig. 6B). At physiological maturity, SLW and SSW of the hybrids were 76.2 g m^−2^ and 829 g m^−3^, which were 29.8% and 20.7% respectively lower than those of the inbred lines (109 g m^−2^ and 1046 g m^−3^).

## Discussion

Physiological processes of N uptake, distribution and remobilization within the plant are closely associated with grain yield formation of maize. With this point of view, genetic improvement aiming at maize grain yield in the past decades has certainly and indirectly influenced nitrogen use efficiency and even unintentionally improved maize heterosis for NUE^11^. Chen, *et al*.^24^ investigated the effect of different N rates on heterosis for NUE of maize inbred lines and their F_1_ crosses. Their results showed that NUE heterosis of maize hybrids was characterized by both mid-parent and over dominance heredity, and was more influenced by the female parent. But in their study, over-standard heterosis (superior performance of F_1_ over the performance of the standard check hybrid) and comparative advantage index (for testing inheritance effects qualitatively) were used to express heterosis for NUE, which was inappropriate to quantify the heterosis for NUE. In another study, the NUE evolution rates of historical cultivars (including landraces, double-crosses and hybrids) and inbred lines of maize from 1950s to 2000s were compared^25^. The NUE evolution rates appeared to be higher in the hybrids than in the inbred lines. However, as landraces and double crosses from early decades were included in their analysis, the results failed to clarify whether there was a heterotic effect of maize hybrids for NUE. To address the above issues, only typical commercial hybrids (XY335 and ZD958; both were widely grown in China Maize Belt) and their parental inbred lines were chosen to evaluate the heterosis for NUE under two N rates. Our study allows for a better understanding of maize heterosis for NUE and more importantly partitioning components of NUE and related traits. The results showed that the absolute heterosis for maize NUE was approximately 13.1 to 24.8 kg grain kg^−1^ N, representing 174% to 422% of the mid-parental heterosis for NUE. This indicates a strong heterosis for NUE of maize hybrids. This MPH value for NUE was obviously higher than that for grain yield reported for the Chinese maize hybrids by Ci, *et al*.^26^, or for the North American maize hybrids by Tollenaar, *et al*.^18^. The reason for this difference may be partly because heterosis for NUE must be judged between no N and N applied treatments. In this case, hybrids responded more sensitively to the applied N treatments than inbred lines did. However, heterosis for grain yield was only examined under the normal N applied conditions in the above cited studies by Ci *et al*.^26^ and Tollenaar, *et al*.^18^.

Tollenaar, *et al*.^18^ previously proposed that the physiological basis of heterosis for grain yield in maize was attributed to the heterosis for leaf size, maximum LAI, leaf stay green and harvest index (HI). The physiological basis underlying heterosis for NUE of maize has not been elucidated until now. Using a meta-analysis approach, Ciampitti and Vyn^11^ pointed out that higher NUE of new era maize cultivars was primarily associated with the reduced grain %N and with the improved NIE. Our current results illustrated that maize hybrids exhibited a strong heterosis for NIE with MHP value of 175% but showed no heterosis for NRE. In this study, the heterosis for NIE accounted for 52.3% of the heterosis for NUE. Since reproductive shoot N remobilization was highly correlated with NIE, efficient reproductive N remobilization served as the important physiological basis for NUE heterosis of maize hybrids.

The proportion of the reproductive remobilized N to grain N varied largely from 45% to 65% among different genotypes and environments^27,28^. Ciampitti and Vyn^29^ suggested that the magnitude of reproductive remobilized N was primarily based on the amount of vegetative N uptake. In this study, comparing N processes between 0 N and the applied N treatment allowed for a better understanding of heterotic effects on N uptake and reproductive N remobilization: (i) although the increments of total N uptake from 0 N to 150 N were similar between hybrids and the inbred lines, the sources of the increment of the hybrids were different from the inbred lines. The N uptake increment was primarily derived from the vegetative N uptake for the hybrids, and mainly originated from the reproductive N uptake of the inbreds. Heterosis for vegetative N uptake of maize hybrids was an important base for efficient reproductive N remobilization; (ii) shoot N remobilization ratio (NRR) and shoot remobilized N (including RLN and RSN) were 10% and 98% greater for the hybrids than for the inbred lines. This induced a 13.4% higher contribution ratio of shoot remobilization N to the grain N in the hybrids than their parental inbred lines; (iii) hybrids had a lower grain %N compared to inbred lines, with similar shoot %N. Taken together, the main physiological basis of heterosis for NIE of maize hybrids could be summarized in two key aspects. Firstly, heterosis for pre-anthesis nitrogen accumulation, and then higher remobilized amount of vegetative N, especially N remobilized from leaf tissues during the reproductive stage. Secondly, heterosis for higher productive efficiency per unit grain nitrogen, which was firstly related to a reduced grain %N in hybrids and then related to heterosis for sink strength (MPH of 49.2% for Kn and of 21.7% for Kw). Hirel and Lemaire^30^ and Ciampitti and Vyn^11^ have previously associated the improved NUE of modern maize genotypes with the reduced grain %N. Our results confirmed that reduced grain %N contributed largely to the heterosis for NIE and also for NUE of maize hybrids. It is noticeable that a “ceiling effect” existed in the relationship of N remobilization and NIE. It appeared that NIE will reach its potential at around 70 kg grain kg^−1^ N ha^−1^, with the remobilized N of 111 kg N ha^−1^. This indicates a trade-off between pre-anthesis N uptake and post-anthesis N remobilization, similar to the trade-off between NRE and NIE, as previously reported by Wang, *et al*.^15^.

Changes of N remobilization are usually accompanied with alterations in plant phenotypic traits. Munaro, *et al*.^31^ previously investigated the heterotic responses of maize grain yield and the related ecophysiological traits to nitrogen availability. They demonstrated that heterosis for grain yield under different N conditions was partly associated with heterosis for radiation use efficiency during grain filling, and with traits related to canopy light capture such as LAI_MAX_. However, these authors failed to examine the relationship between NUE and the related phenotypic parameters in their study. Ciampitti, *et al*.^16^ found that leaf chlorophyll (SPAD readings) and plant stem volume at silking stage were highly correlated to final plant N uptake, grain yield and NUE of maize hybrids, but they did not explore the association between heterosis for NUE and the related phenotypic changes. The results of our present study illustrated that both quantitative and qualitative phenotypic traits of maize canopy influenced the reproductive N remobilization. (i) With respect to source establishment, leaf appearance rate (RLA), LAI_R1_, chlorophyll content (SPAD value) and photosynthetic nitrogen use efficiency (PNUE) during the vegetative stage were 9.9%, 88%, 12.5% and 68.7%, respectively superior for hybrids than for the inbred lines. This supported the greater vegetative N uptake of hybrids and then sufficient N supply for the reproductive remobilization. (ii) With respect to source senescence and induced N remobilization from leaves and stalks, there was 26% more reduction in LAI, 29.8% lower specific leaf weight (SLW) and 20.7% lower specific stalk weight (SSW), respectively for hybrids than for their inbred lines. Therefore, additional phenotyping of leaf number, leaf area, biomass of vegetative organs (leaves and stalk) and volume of stalk should be necessary to get the mechanistic understanding of NIE and NUE. In this point of view, more research on NUE related phenotypes and heterotic prediction based on NUE phenotyping is warranted.

## Conclusions

Our study revealed a strong heterosis for NUE of maize hybrids, which was primarily derived from heterosis for NIE. Heterosis for NIE was ascribed to (i) an earlier establishment of pre-anthesis source for nitrogen accumulation. This was phenotypically associated with faster leaf appearance rate, higher maximum LAI and photosynthetic nitrogen use efficiency; (ii) a higher nitrogen remobilization of vegetative organs, especially nitrogen remobilized from leaves during the reproductive stage. This was phenotypically exhibited in a faster leaf area recession, a lower specific weight of leaf and stalk, and maintaining functional stay-green of ear leaves; and (iii) a higher productive efficiency per unit grain nitrogen. This was characterized by a reduced grain N concentration and enhanced sink strength. Therefore, phenotypic exploration and isolation of related gene(s) of both the source and the sink should be warranted for the utilization of heterosis for NUE in maize.

## Materials and Methods

### Experimental site

A field experiment was conducted for two years (2014 and 2015) under an irrigated cropping system at a farm near Tumed Right Banner County, Inner Mongolia, China (40°33′N, 110°31′E, 996 m a.s.l.). The test site was located in a typical northwest China maize production region, with semi-arid continental monsoon climate. The total precipitation during the two growing seasons were 358.5 mm and 423.1 mm, respectively. Irrigation was used to maintain the soil water content at ≥75% of the field capacity.

The experimental field had a sandy loam soil texture with continuous maize (Zea mays L.) production prior to the current study. In order to replicate this experiment with same previous crop condition, the experimental fields in 2014 and 2015 were changed. Before land preparation, composite soil samples (0–30 cm depth) were taken and analyzed. The soil contained 24.5 g kg^−1^ organic matter, 21.2 mg kg^−1^ alkali-hydrolyzable N, 26.7 mg kg^−1^ Olsen-P (NaHCO_3_ extractant), and 120.4 mg kg^−1^ soil test K (NH_4_OAc-K) with a pH of 7.5 in 2014, and 16.5 g kg^−1^ organic matter, 19.8 mg kg^−1^ alkali-hydrolyzable N, 16.5 mg kg^−1^ Olsen-P, 90.4 mg kg^−1^ soil test K and a pH of 7.9 in 2015. To ensure adequate plant growth, 75 kg ha^−1^ P_2_O_5_, 30 kg ha^−1^ N, (Diammonium phosphate, contain 46% P_2_O_5_ and 18% N), and 45 kg ha^−1^ K_2_O (Potassium sulphate, contain 50% K_2_O) were applied each year as the starter fertilizer which was incorporated into 0–15 cm of the soil with rotary tillage before planting.

### Experimental design

Two typical and widely-grown commercial maize hybrids Xianyu335 (XY335) and Zhengdan958 (ZD958), and their parental inbred lines (PH4CV and PH6WC for XY335; Zheng58 and Chang7-2 for ZD958) were grown at two N rates, 0 (0 N) and 150 kg N ha^−1^ (150 N). The applied N was side-dressed at the 6-leaf stage. The experiment was arranged in a randomized complete block design with four replications. Each plot was 5 m long and consisted of 10 rows of maize spaced 0.6 m apart. A 1-m gap was used to separate the blocks. The plant density was 75 000 plants ha^−1^. Complete weed control was obtained with pre-plant and post-emergence herbicides. In both years, 50 mm of irrigation water at the V10 growth stage and another 50 mm at the R1 stage were supplied through flood irrigation. Pesticides were sprayed by unmanned aerial vehicle when needed.

### Sampling procedures

After seedling emergence, 30 consecutive plants with proper borders and uniform plant-to-plant spaces were tagged in each plot for morpho-physiological measurements. Maize phenology was tracked from V6 to R6 (VE, V6, V14, R1, R3 and R6) on the tagged plants, and the total leaf number was measured nondestructively at each phenological stage. Plant height (PH) (measured from the stem base to the uppermost developed leaf tip) and maximum stalk diameter (STD) were determined at silking (R1) and at maturity (R6) stages. The STD was measured with a Mitutoyo Absolute Digimatic caliper (Mitutoyo China Corporation, Shanghai, China) at the middle section of the sixth internode. At silking, 5 consecutive plants from a designated area were selected for SPAD and leaf photosynthesis measurements. The SPAD measurements were determined on the ear leaf of each plant and averaged, by using the Konica Minolta SPAD-502Plus Chlorophyll Meter (Konica Minolta China Investment Ltd., Shanghai, China). Leaf apparent photosynthetic rate (Pn) was measured on ear leaf using a Li-6400 portable open-flow photosynthesis system (Li-Cor Inc., Lincoln, NE, USA). During the measurements, temperature inside the leaf cuvette was set to 25 °C, relative humidity was adjusted to near ambient level, the photosynthetic photon flux density (provided by a red/blue LED light source, 6400-02 LED) was set at 1500 µmol m^−2^ s^−1^. The carbon dioxide (CO_2_) concentration in the cuvette was maintained at 360 µL L^−1^.

Total aboveground biomass was sampled for the determination of BM and N uptake at R1 (silking) and R6 (physiological maturity) stages. At V6 stage, another 20 consecutive and uniform plants were tagged in each plot for BM sampling in order to avoid inconsistency plants. At each sampling, 5 consecutive plants from the tagged area were cut at the stem base, separated into leaves, stalks (including stems, leaf sheaths and tassels) and ear BM (husk and cobs) at the R1 stage, and into leaves, stalks (including stems, leaf sheaths, tassels, ear shanks, husks and cobs) and grain BM at the R6 stage. Prior to chopping, green leaf area was determined by measuring the leaf length (L) and the maximum leaf width (W) of each leaf, and calculated, according to the formulae L × W × 0.75. LAI values were also calculated. The chopped plant samples were dried at 85 ± 5 °C to constant weight for recording the dry weights. Dried plant material was ground for the determination of N concentration, using the Kjeldahl method. The N content of each fraction was calculated as the product of N concentration by its biomass.

At physiological maturity (R6), all ears from the central two rows of each plot were hand-harvested. After the kernel number per ear was determined, the ear was shelled and recorded for grain moisture, total biomass and grain weight. Grain yield was calculated and reported on a 140 g kg^−1^ water basis. The harvest index (HI) was calculated as the ratio of the grain weight (0% moisture) to the total aboveground plant BM.

### Nitrogen and heterosis indices

For each genotype of maize receiving N application, NUE was calculated as the ratio of incremental grain yield response (yield of N fertilized plots – yield of the unfertilized plots) to the N fertilizer applied^32^. Other nitrogen utilization parameters, including nitrogen recovery efficiency, nitrogen internal efficiency (NIE), nitrogen harvest index, shoot N remobilization ratio (NRR) and contribution ratio of shoot remobilization N to grain N were also calculated, according to Ciampitti and Vyn^33,34^:

1 $${\rm{NUE}}=({{\rm{GY}}}_{{\rm{fert}}{\rm{.}}}-{{\rm{GY}}}_{{\rm{unfert}}{\rm{.}}})/{\rm{N}}\,{\rm{fertilizer}}\,\mathrm{applied},$$

where GY_fert._ is the grain yield (kg ha^−1^ at 14% grain moisture) of a treatment receiving 150 kg N ha^−1^ fertilizer, and GY_unfert._ is the grain yield of 0 N treatment.

2 $${\rm{NRE}}=({{\rm{Nupt}}}_{{\rm{fert}}{\rm{.}}}-{{\rm{Nupt}}}_{{\rm{unfert}}{\rm{.}}})/{\rm{N}}\,{\rm{fertilizer}}\,\mathrm{applied},$$

3 $${\rm{NIE}}=({{\rm{GY}}}_{{\rm{fert}}{\rm{.}}}-{{\rm{GY}}}_{{\rm{unfert}}{\rm{.}}})/({{\rm{Nupt}}}_{{\rm{fert}}{\rm{.}}}-{{\rm{Nupt}}}_{{\rm{unfert}}{\rm{.}}}),$$

where Nupt_fert._ is N uptake in the 150 kg N ha^−1^ treatment and Nupt_unfert._ is N uptake in the 0 kg N ha^−1^ treatment.

4 $${\rm{NHI}}={\rm{Grain}}\,{\rm{N}}/({\rm{Shoot}}\,{\rm{N}}+{\rm{Grain}}\,{\rm{N}})\times \mathrm{100} \% ,$$

where the shoot N fraction includes leaf, stem, leaf sheath, cob, shank and husk components.

5 $${\rm{NRR}}=({\rm{Shoot}}\,{\rm{N}}\,{\rm{at}}\,{\rm{silking}} \mbox{-} {\rm{Shoot}}\,{\rm{N}}\,{\rm{at}}\,{\rm{maturity}})/{\rm{Shoot}}\,{\rm{N}}\,{\rm{at}}\,{\rm{silking}}\times \mathrm{100} \% ,$$

6 $${\rm{NCR}}=({\rm{Shoot}}\,{\rm{N}}\,{\rm{at}}\,{\rm{silking}} \mbox{-} {\rm{Shoot}}\,{\rm{N}}\,{\rm{at}}\,{\rm{maturity}})/{\rm{Grain}}\,{\rm{N}}\times \mathrm{100} \% ,$$

Photosynthetic nitrogen-use efficiency (PNUE) was calculated as the ratio of Pn to leaf N content per unit leaf area^35^,

7 $${\rm{PNUE}}={\rm{Pn}}/{\rm{leaf}}\,{\rm{N}}\,{\rm{content}}\,{\rm{per}}\,{\rm{unit}}\,{\rm{leaf}}\,\mathrm{area},$$

where Pn and leaf N content per unit leaf area are all measured at the R1 stage.

In order to systematically compare the difference between hybrids and their parental inbred lines, both absolute heterosis (AH) and mid-parent heterosis (MPH) were determined in this study. MPH, which measures the superior performance of a hybrid over the mean performance of its parental inbred lines, was calculated as:

8 $${\rm{MPH}}=[({{\rm{F}}}_{{\rm{1}}}\,-\,{\rm{MP}})/{\rm{MP}}]\times \mathrm{100},$$

where F_1_ is the measure of the hybrid performance and MP = (P_1_ + P_2_)/2, where P_1_ and P_2_ are the performance values of the parental inbred lines, respectively^18^. (F_1_ − MP) is the AH, which means the difference of the specific index between the F_1_ hybrid and the mid-parent value^22^.

### Statistical analysis

Analysis of variance was executed using the PROC MIXED procedure of SPSS 17.0 software (SPSS Institute Inc., USA), where genotype and N treatments were considered fixed factors, and year and blocks were random factors. We combined data from hybrids or inbred lines separately, since for most variables, no significant differences were found between the two hybrids or among the four inbred lines. Because the error mean squares are found to be heterogeneous for most variables measured, the results of individual years are presented separately. Comparisons among different treatments were based on an ANOVA-protected LSD test at the 0.05 level of probability. To quantify the variance of heterosis for NUE, the proportion of the variance (quantified by the r^2^ values) was accounted for by linear regressions between mid-parental heterosis values of NUE and its component parameter (MPH of NRE and MPH of NIE). This was similar to the procedure used by Duvick and Cassman^36^ and Ciampitti and Vyn^29^. The “linear + plateau” relationship of remobilized N and NIE was established by a nonlinear procedure of SPSS 17.0 software, and the correlation analysis was also executed using the Bivariate Correlation procedure of SPSS 17.0 software.
